# Supplementary material for: Identification of a pyrophosphate-dependent kinase and its donor selectivity determinants
Source: Nat Commun. 2018 May 2;9:1765. doi: 10.1038/s41467-018-04201-z (PMC5931981; doi:10.1038/s41467-018-04201-z)
Supplement: Supplementary file 1 — Supplementary Information [file 41467_2018_4201_MOESM1_ESM.pdf]

Supplementary Information

Identification of a pyrophosphate-dependent kinase and its donor selectivity determinants

Nagata *et al.*

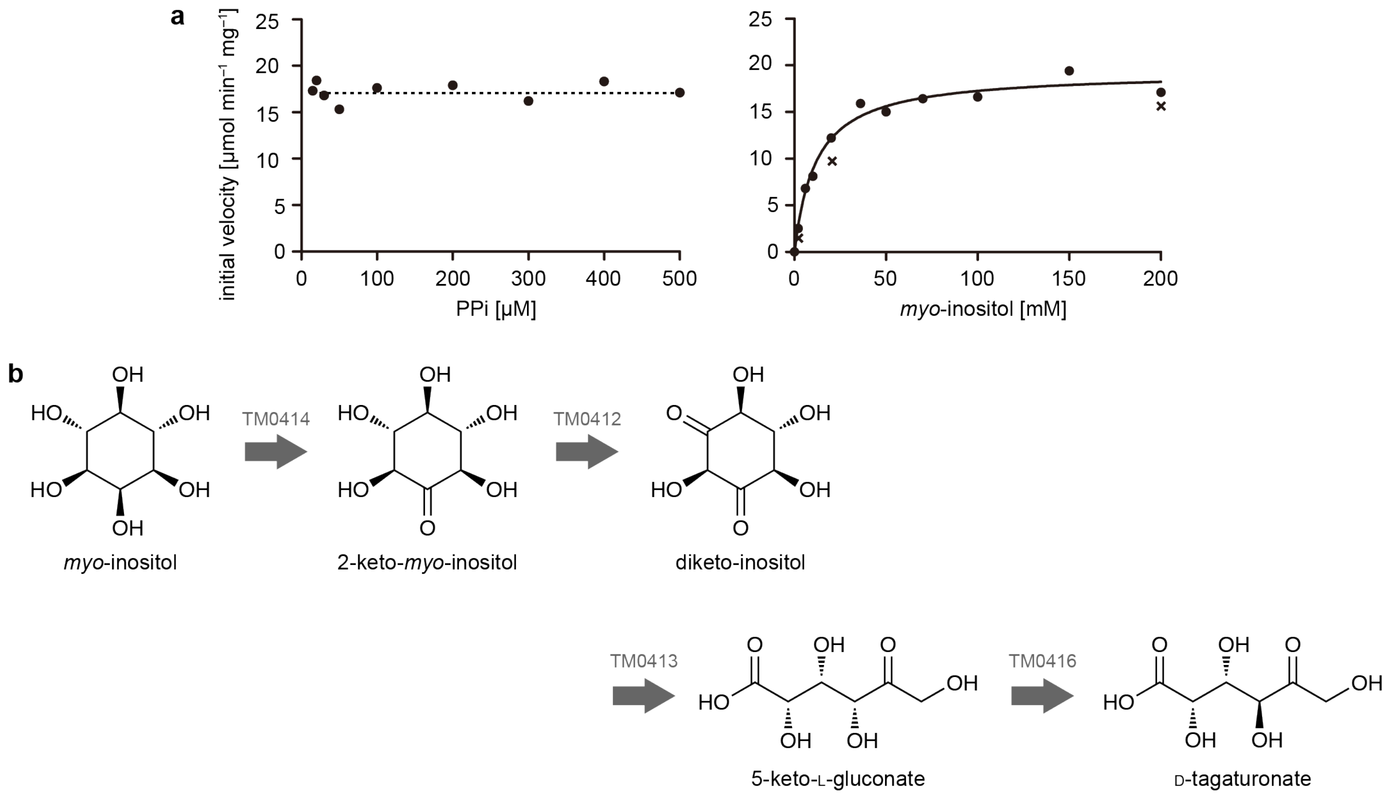

Supplementary Figure 1. Kinetic analysis and potential phosphate acceptors of TM0415 (**a**) Kinetic analysis of the wild-type TM0415. The vertical and horizontal axes represent the initial velocity of the reaction and the concentrations of the substrates, respectively. The dotted line in the left panel represents an average value of the initial velocities, which was used for calculating the k_{cat} value toward PPi. Closed circles represent the initial velocities measured without KCl, while x marks (only in the right panel) represent the initial velocities in the presence of 100 mM KCl. The data were obtained from single measurements. (**b**) *myo*-Inositol metabolic pathway composed of enzymes encoded in the TM0411–TM0416 operon in *Thermotoga maritima* [1]. One of the compounds in this pathway might be the genuine acceptor of TM0415.

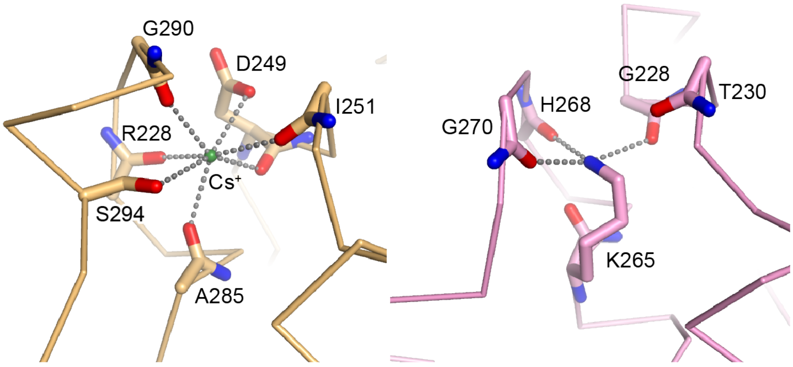

Supplementary Figure 2. Structural comparison of the monovalent-cation-binding site. The cation-binding site of ribokinase from *Escherichia coli* (Protein Data Bank (PDB) ID 1GQT) [2] and the unliganded TM0415 (1VK4) are shown in the left and right panels, respectively. Gray dotted lines show the interactions involving the cesium ion and K265. Residues and the cesium ion are shown using stick and green sphere models, respectively.

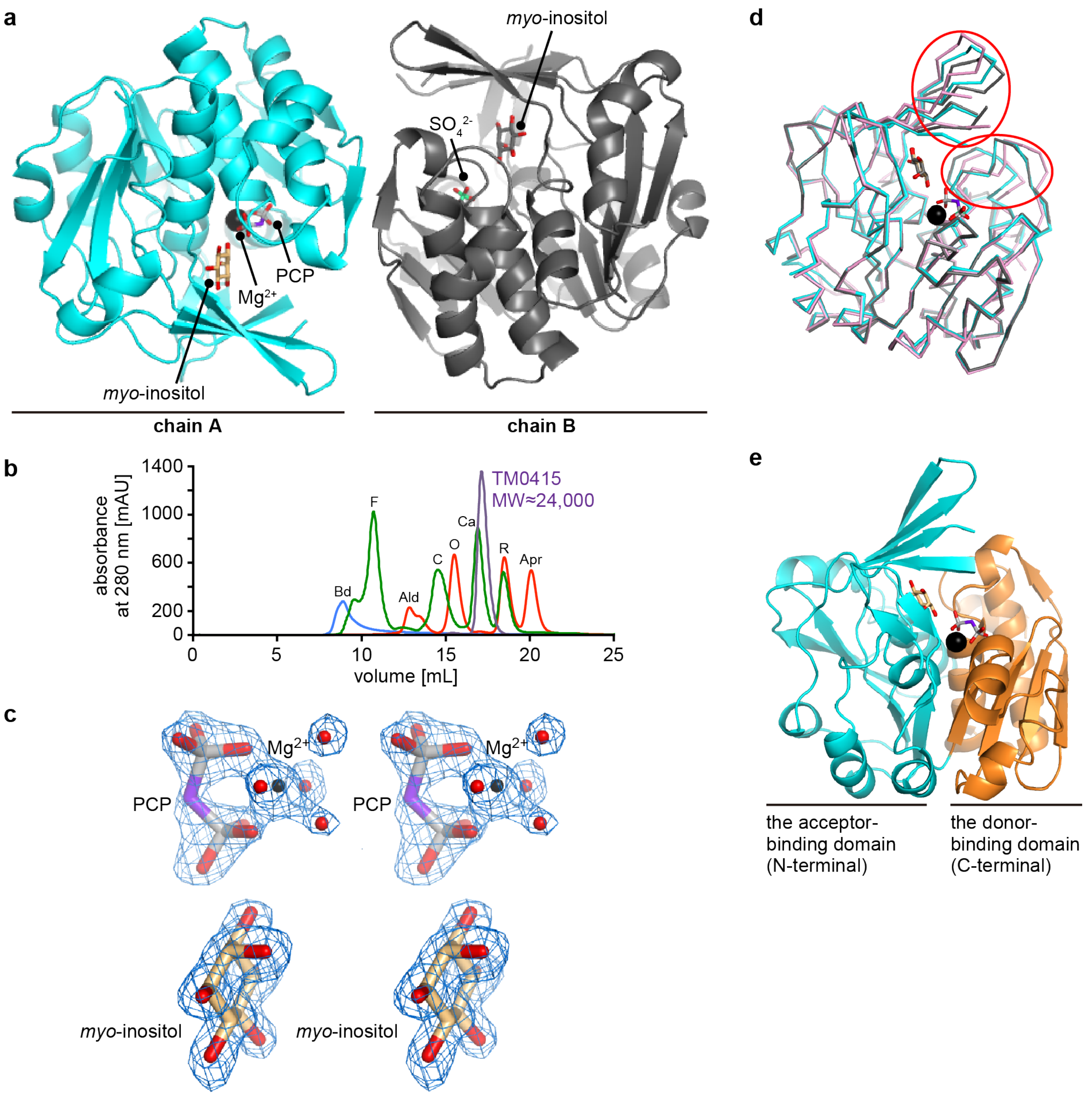

Supplementary Figure 3. Overall structures and oligomeric assembly analysis of TM0415. (**a**) Chains A (cyan) and B (gray) in an asymmetric unit of the PCP-complex crystal. Chain A is bound to PCP, *myo*-inositol, and a magnesium ion, while chain B contains a sulfate ion instead of PCP and the magnesium ion. (**b**) Investigation of oligomeric assembly. The purple line indicates the elution volume of the TM0415 recombinant protein. Based on the elution volume, the molecular weight in solution was estimated. Abbreviations of marker protein names; Bd, blue dextran (theoretical molecular weight 2,000k); F, ferritin (440k); Ald, aldolase (158k); C, conalbumin (75k); O, ovalbumin (44k); Ca, carbonic anhydrase (29k); R, ribonuclease A (13.7k); Apr, aprotinin (6.5k). (**c**) Stereo view of the F_o–F_c omit map for the ligands in chain A contoured at 3.5 σ. (**d**) Superposition of chains A and B on the unliganded structure (pink, PDB ID 1VK4). Red ellipses indicate the regions that show slight differences among the three structures. (**e**) The two domains observed in the ribokinase family enzymes. The acceptor-binding and donor-binding domains correspond to the N-terminal and C-terminal halves of the enzymes, respectively. The donor-binding domain in the TM0415 structure (the C-terminal residues 169–286) is colored orange. The Cα traces are shown using a cartoon model in panels a and e. PCP, the sulfate ion, and *myo*-inositol are shown as sticks. The magnesium ion and water molecules are represented as black and red spheres, respectively.

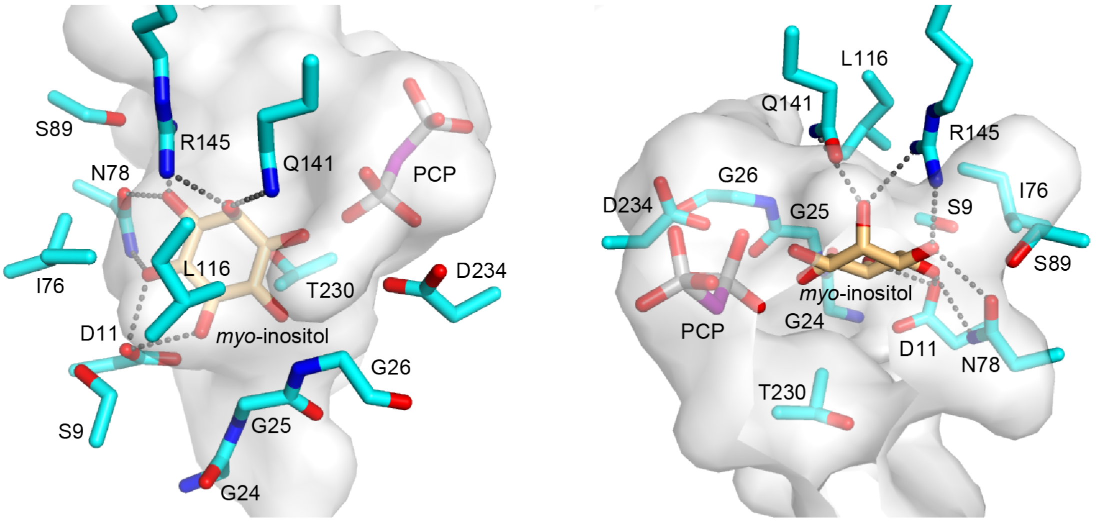

Supplementary Figure 4. Residues around *myo*-inositol (within 4.5 Å) in TM0415. *myo*-Inositol, PCP, and the residues around the ligands are shown as stick models. The gray envelope represents the *myo*-inositol-binding pocket. Gray dotted lines show the interactions between *myo*-inositol and the residues. The two panels are drawn from different viewpoints.

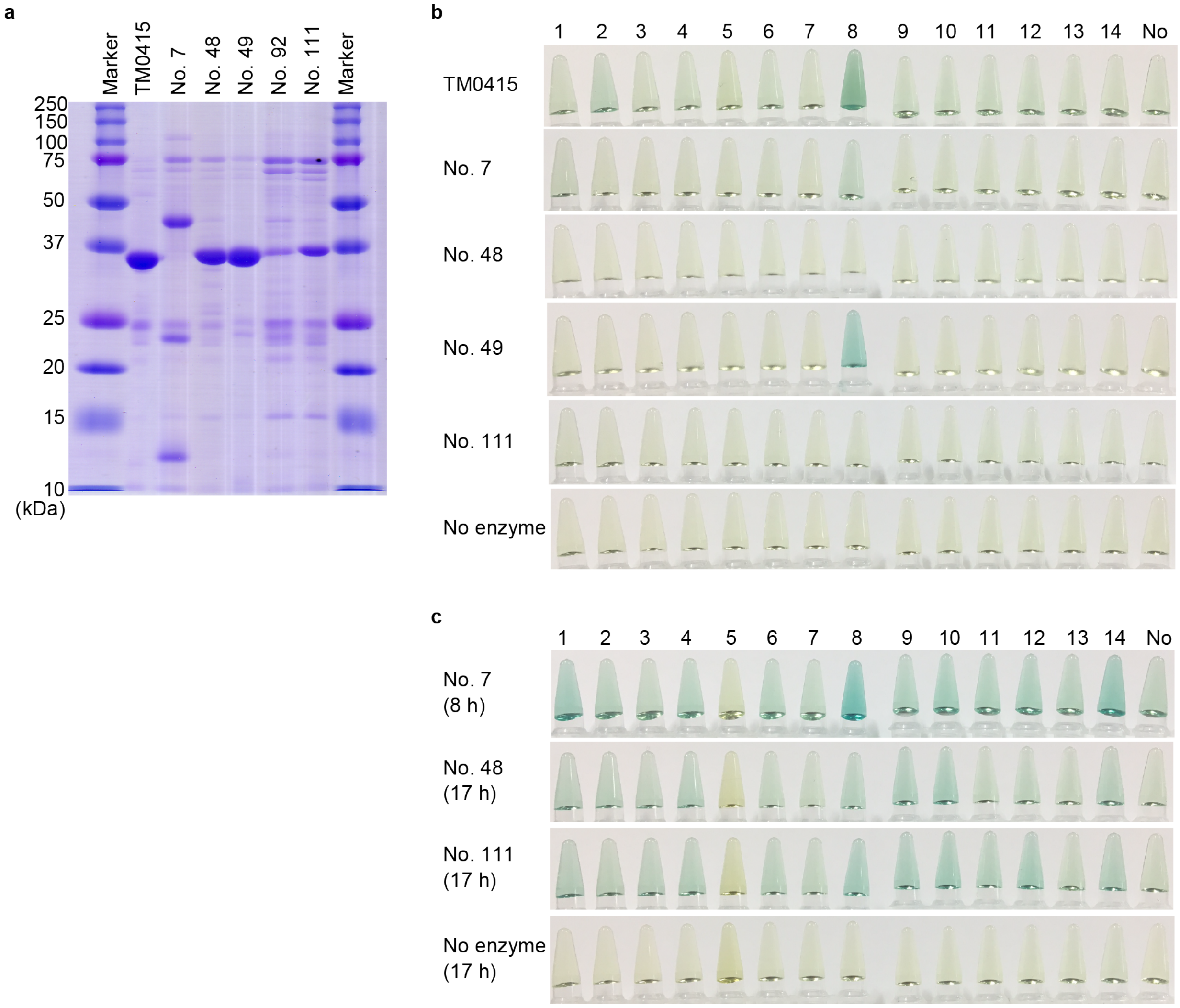

Supplementary Figure 5. Analyses of the TM0415 homologs. (**a**) SDS-PAGE analysis on the homologs after partial purification. The molecular weight of homolog No. 7 is about 45,000, and those of the other proteins are approximately 34,000. (**b, c**) Analyses of the PPi-dependent activity by the malachite green assay. The enzymatic reaction was performed for 10 min (**b**) and 8 or 17 h (**c**). The depth of blue color of the mixture represents the amount of Pi produced by the enzymatic reaction from PPi. The numbers above the pictures correspond to the following acceptors: 1, D-ribose; 2, D-xylose; 3, D-fructose; 4, D-glucose; 5, D-glucosamine; 6, glycerol; 7, *meso*-erythritol; 8, *myo*-inositol; 9, sucrose; 10, maltose; 11, inosine; 12, adenosine; 13, cytidine; 14, 2-keto-3-deoxygluconate. “No” means no acceptor.

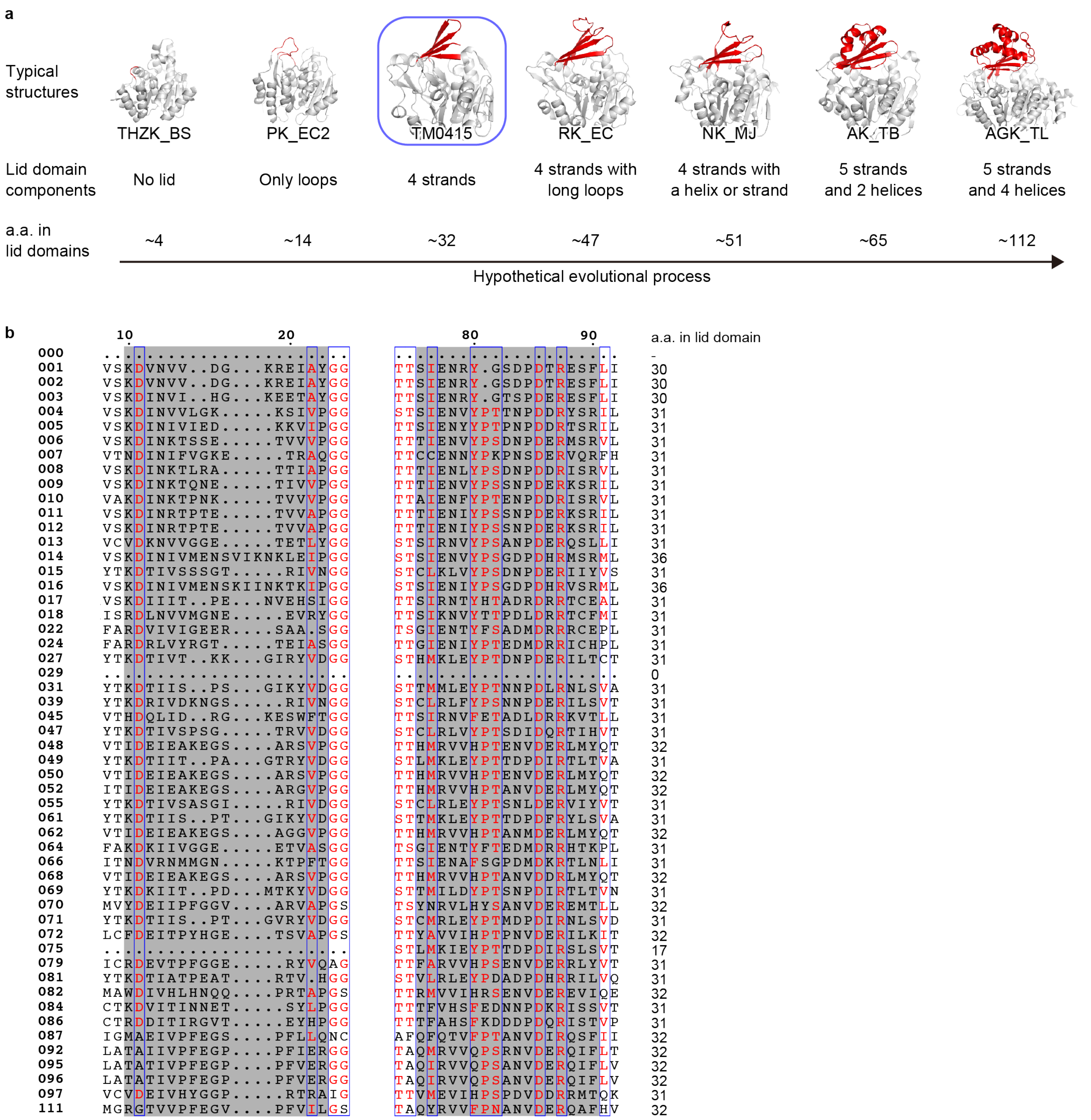

Supplementary Figure 6. Comparison of the lid domain in the ribokinase family enzymes. (**a**) Classification of the ribokinase family enzymes based on the size of the lid domain. This classification is thought to be involved in the evolutional trace of these enzymes [3]. Typical structures in the ribokinase family are shown in gray cartoon models, and their lid domains are highlighted in red. The TM0415 structure is highlighted by a blue box. Abbreviations of the enzymes are shown in **Supplementary Table 7**. (**b**) Sequence alignment of the candidate PPi-dependent kinases. The regions corresponding to the lid domain are highlighted with gray backs. The ID numbers correspond to the numbers in **Supplementary Table 1 and 2**.

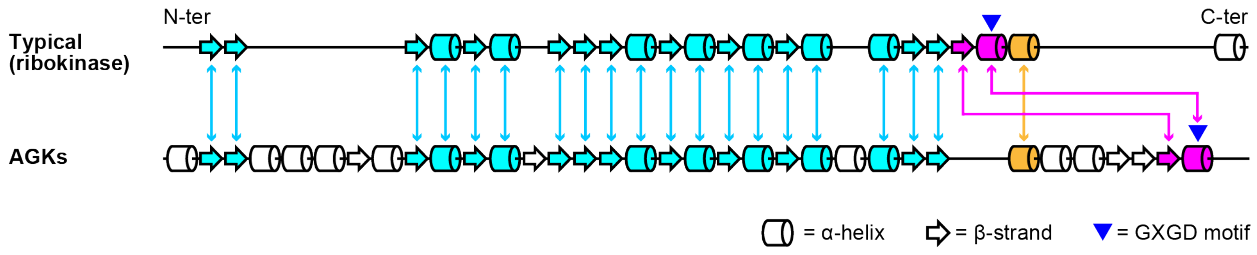

Supplementary Figure 7. Different order of the secondary structures around the GXGD motif between a typical ribokinase and ADP-dependent glucokinases. The order of secondary structure elements for a typical enzyme of the ribokinase family (ribokinase from *E. coli*) and for ADP-dependent glucokinases (AGKs) from *Pyrococcus furiosus* and *Thermococcus litoralis* are represented. Superimposable elements in 3D-structure are colored, and double headed arrows indicate the superimposable element pairs. Blue triangles show locations of the GXGD motif. Two magenta elements in the typical enzyme are located before the helix colored orange, while the corresponding elements in AGKs are located after the orange α-helix. The difference in the order of the magenta and orange elements prevents primary sequence alignment, because primary sequence alignment programs adjust the conserved amino-acid residues by gap insertions, but such insertions cannot change the order of the elements. Thus, residues around the GXGD motif in AGKs are shown in the green box in **Fig. 3d**.

Supplementary Table 1. Candidate PPi-dependent kinases considered to phosphorylate the same acceptor as that of TM0415.

	Conservation of the residues																				
	Interacting with PPi			At the ATP-binding pocket		Interacting with *myo*-inositol				Positioned near to *myo*-inositol (4.5 Å)											
No.	K171	R229	R232	F221	M266	D11	N78	Q141	R145	S9	G24	G25	G26	I76	S89	L116	T230	D234	Accesssion	E-value	Description	Pylum
0	K	R	R	F	M	-	-	-	-	-	-	-	-	-	-	-	T	D			169–286 residues of TM0415	Thermotogae
1	K	R	R	F	M	D	N	Q	R	S	G	G	G	I	S	L	T	D	WP_004083266.1	5E-79	carbohydrate kinase [Thermotoga maritima]	Thermotogae
2	K	R	R	F	SEM	D	N	Q	R	S	G	G	G	I	S	L	T	D	1VK4_A	2E-76	Chain A, Crystal Structure Of Pfkb Carbohydrate Kinase (tm0415) From Thermotoga Maritima At 1.91 A Resolution	Thermotogae
3	K	R	R	F	M	D	N	Q	R	S	G	G	G	I	S	L	T	D	WP_008191991.1	2E-55	MULTISPECIES: carbohydrate kinase [Thermotoga]	Thermotogae
4	K	R	R	F	M	D	N	Q	R	S	G	G	G	I	S	L	T	D	WP_015868351.1	5E-33	MULTISPECIES: carbohydrate kinase [Kosmotoga]	Thermotogae
5	K	R	R	F	M	D	N	Q	R	S	G	G	G	I	S	L	T	D	WP_047754545.1	1E-31	carbohydrate kinase [Kosmotoga pacifica]	Thermotogae
6	K	R	R	F	M	D	N	Q	R	S	G	G	G	I	S	L	T	D	WP_041078582.1	9E-28	hypothetical protein [Thermotoga caldifontis]	Thermotogae
8	K	R	R	F	M	D	N	Q	R	S	G	G	G	I	S	L	T	D	WP_013932391.1	2E-27	hypothetical protein [Pseudothermotoga thermarum]	Thermotogae
9	K	R	R	F	M	D	N	Q	R	S	G	G	G	I	S	L	T	D	KUK02818.1	3E-27	hypothetical protein XD45_1042 [Thermotoga sp. 50_64]	Thermotogae
10	K	R	R	F	M	D	N	Q	R	A	G	G	G	I	S	L	T	D	WP_041084097.1	9E-27	carbohydrate kinase [Thermotoga profunda]	Thermotogae
11	K	R	R	F	M	D	N	Q	R	S	G	G	G	I	S	L	T	D	WP_031505061.1	4E-26	hypothetical protein [Pseudothermotoga hypogea]	Thermotogae
12	K	R	R	F	M	D	N	Q	R	S	G	G	G	I	S	L	T	D	AJC74166.1	4E-26	carbohydrate kinase [Pseudothermotoga hypogea DSM 11164 = NBRC 106472]	Thermotogae
13	K	R	R	F	M	D	N	Q	R	C	G	G	G	I	S	L	T	D	OHD67114.1	1E-24	hypothetical protein A2177_15780 [Spirochaetes bacterium RBG_13_68_11]	Spirochetes
14	K	R	R	F	M	D	N	Q	R	S	G	G	G	I	S	L	T	D	WP_047265272.1	5E-23	carbohydrate kinase [Marinitoga sp. 1155]	Thermotogae
16	K	R	R	F	M	D	N	Q	R	S	G	G	G	I	S	L	T	D	WP_072862509.1	7E-22	carbohydrate kinase [Marinitoga hydrogenitolerans]	Thermotogae
17	K	R	R	F	M	D	N	Q	R	S	G	G	A	I	C	L	T	D	OQC03565.1	9E-21	pfkB family carbohydrate kinase [Candidatus Hydrogenedentes bacterium ADurb.Bin101]	(Bacteria)
18	K	R	R	F	M	D	N	Q	R	S	G	G	G	I	C	L	T	D	GAK58449.1	1E-20	PfkB domain protein [Candidatus Vecturithrix granuli]	(Bacteria)
22	K	R	R	F	M	D	N	Q	R	A	G	G	A	I	C	I	T	D	OGR03017.1	5E-20	hypothetical protein A2284_03830 [Deltaproteobacteria bacterium RIFOXYA12_FULL_61_11]	Proteobacteria
24	K	R	R	F	M	D	N	Q	R	A	G	G	G	I	C	L	T	D	OFX28282.1	2E-19	hypothetical protein A2Z07_08620 [Armatimonadetes bacterium RBG_16_67_12]	Armatimonadetes
45	K	R	R	F	M	D	N	Q	R	T	G	G	G	I	V	L	T	D	OHD72463.1	2E-16	hypothetical protein A2V99_02430 [Spirochaetes bacterium RBG_16_67_19]	Spirochetes
64	K	R	R	F	M	D	N	Q	R	A	G	G	S	I	T	I	T	D	KPL23139.1	4E-15	hypothetical protein AMJ93_05390 [Anaerolineae bacterium SM23_84]	Chloroflexi

The ID numbers correspond to the order of the E-values in 143 homologs found by the BLAST search, which include the 52 proteins possessing the five key residues (No. 0 is the submitted sequence, the sequence of No. 1 displayed the smallest E-value, and the sequence of No. 143 displayed the largest E-value). Conserved residues in the donor-binding site are highlighted in blue or gray. Domains of life are written in parentheses if the bacterial phylum is not defined.

Supplementary Table 2. Candidate PPi-dependent kinases considered to phosphorylate a different acceptor from that of TM0415.

	Conservation of the residues																					
	Interacting with PPi			At the ATP-binding pocket		Interacting with *myo*-inositol				Positioned near to *myo*-inositol (4.5 Å)												
No.	K171	R229	R232	F221	M266	D11	N78	Q141	R145	S9	G24	G25	G26	I76	S89	L116	T230	D234	Accesssion	E-value	Description	Pylum
7	K	R	R	F	M	D	N	Q	R	T	G	G	G	C	Q	L	T	D	XP_004352863.1	1E-27	ribokinase, putative [Acanthamoeba castellanii str. Neff]	(Eukaryota)
15	K	R	R	F	M	D	L	Q	R	T	G	G	A	L	I	S	S	D	KQC11084.1	3E-22	hypothetical protein APR54_11295 [Candidatus Cloacimonas sp. SDB]	(Bacteria)
27	K	R	R	F	M	D	L	Q	R	T	G	G	G	M	L	S	S	D	OGU60004.1	1E-18	hypothetical protein A2V66_06695 [Ignavibacteria bacterium RBG_13_36_8]	Chlorobi
29	K	R	R	F	M	-	-	-	-	-	-	-	-	-	-	-	T	D	OQB32101.1	2E-18	hypothetical protein BWY09_02890 [Candidatus Hydrogenedentes bacterium ADurb.Bin179]	(Bacteria)
31	K	R	R	F	M	D	L	Q	R	T	G	G	A	M	L	S	S	D	OGO14342.1	5E-18	hypothetical protein A2Y53_06675 [Chloroflexi bacterium RBG_16_47_49]	Chloroflexi
39	K	R	R	F	M	D	L	Q	R	T	G	G	A	L	L	S	S	D	OQC71674.1	6E-17	hypothetical protein BWX45_01133 [Deltaproteobacteria bacterium ADurb.Bin002]	Proteobacteria
47	K	R	R	F	M	D	L	Q	R	T	G	G	A	L	I	S	S	D	KPL23130.1	3E-16	hypothetical protein AMJ93_05395 [Anaerolineae bacterium SM23_84]	Chloroflexi
48	K	R	R	F	M	D	V	Q	R	T	G	G	A	M	M	L	T	D	OPX96711.1	3E-16	aminoimidazole riboside kinase [Syntrophorhabdus sp. PtaB.Bin006]	Proteobacteria
49	K	R	R	F	M	D	L	Q	R	T	G	G	G	M	L	S	S	D	WP_062419767.1	4E-16	hypothetical protein [Levilinea saccharolytica]	Chloroflexi
50	K	R	R	F	M	D	V	Q	R	T	G	G	A	M	M	L	T	D	OPY74118.1	4E-16	aminoimidazole riboside kinase [Syntrophorhabdus sp. PtaU1.Bin002]	Proteobacteria
52	K	R	R	F	M	D	V	Q	R	T	G	G	A	M	M	L	T	D	OPY66382.1	6E-16	pfkB family carbohydrate kinase [Syntrophorhabdus sp. PtaU1.Bin050]	Proteobacteria
55	K	R	R	F	M	D	L	Q	R	T	G	G	A	L	I	S	S	D	OGD17089.1	7E-16	hypothetical protein A2V47_06395 [Candidatus Atribacteria bacterium RBG_19FT_COMBO_35_14]	(Bacteria)
61	K	R	R	F	M	D	L	Q	R	T	G	G	A	M	L	S	S	D	OGO65182.1	2E-15	hypothetical protein A2029_16545 [Chloroflexi bacterium RBG_19FT_COMBO_47_9]	Chloroflexi
62	K	R	R	F	M	D	V	Q	R	T	G	G	A	M	M	L	T	D	WP_051409333.1	2E-15	hypothetical protein [Syntrophorhabdus aromaticivorans]	Proteobacteria
66	K	R	R	F	M	D	N	Q	R	T	G	G	A	I	L	L	V	D	WP_016524852.1	4E-15	hypothetical protein [Treponema maltophilum]	Spirochaetes
68	K	R	R	F	M	D	V	Q	R	T	G	G	A	M	M	L	T	D	OPY81634.1	7E-15	aminoimidazole riboside kinase [Syntrophorhabdus sp. PtaU1.Bin153]	Proteobacteria
69	K	R	R	F	M	D	L	Q	R	T	G	G	A	M	L	S	S	D	OJX43988.1	8E-15	hypothetical protein BGO78_03265 [Chloroflexi bacterium 44-23]	Chloroflexi
70	K	R	R	F	M	D	V	Q	R	V	G	S	A	N	M	I	T	D	OQB78664.1	1E-14	hypothetical protein BWX87_02598 [Bacteroidetes bacterium ADurb.Bin123]	(Bacteria)
71	K	R	R	F	M	D	L	Q	H	T	G	G	A	M	L	S	S	D	OIO83792.1	1E-14	hypothetical protein AUK02_07515 [Anaerolineae bacterium CG2_30_58_95]	Chloroflexi
72	K	R	R	F	M	D	V	Q	R	C	G	S	A	A	L	I	T	D	GAK58928.1	1E-14	PfkB domain protein [Candidatus Vecturithrix granuli]	(Bacteria)
75	K	R	R	F	M	-	I	Q	R	-	-	-	-	M	L	S	S	D	OGO66208.1	3E-14	hypothetical protein A2Z45_06290 [Chloroflexi bacterium RBG_19FT_COMBO_55_16]	Chloroflexi
79	K	R	R	F	M	D	V	Q	R	C	A	G	S	A	L	I	T	D	OPZ84176.1	7E-14	hypothetical protein BWY76_01960 [bacterium ADurb.Bin429]	(Bacteria)
81	K	R	R	F	M	D	L	Q	R	T	G	G	A	L	I	S	S	D	OHD27353.1	1E-13	hypothetical protein A2064_11710 [Spirochaetes bacterium GWB1_66_5]	Spirochetes
82	K	R	R	F	M	D	V	Q	R	A	G	S	A	M	V	M	T	D	OGJ92064.1	4E-13	hypothetical protein A2268_05905 [Candidatus Raymondbacteria bacterium RifOxyA12_full_50_37]	(Bacteria)
84	K	R	R	F	M	D	H	Q	R	T	G	G	G	F	S	L	T	D	XP_001320377.1	7E-13	hypothetical protein [Trichomonas vaginalis G3]	(Eukaryota)
86	K	R	R	F	M	D	H	Q	R	T	G	G	G	F	S	L	T	D	OHT03327.1	1E-12	hypothetical protein TRFO_29306 [Tritrichomonas foetus]	(Eukaryota)
87	K	R	R	F	M	A	T	Q	L	G	N	C	S	F	S	M	M	D	OPY65922.1	1E-12	pfkB family carbohydrate kinase [Syntrophorhabdus sp. PtaU1.Bin050]	Proteobacteria
92	K	R	R	F	M	A	V	Q	W	A	G	G	P	M	I	L	T	D	WP_051408910.1	4E-11	carbohydrate kinase family protein [Syntrophorhabdus aromaticivorans]	Proteobacteria
95	K	R	R	F	M	A	V	Q	W	A	G	G	P	I	I	L	T	D	OPY67903.1	2E-10	pfkB family carbohydrate kinase [Syntrophorhabdus sp. PtaU1.Bin002]	Proteobacteria
96	K	R	R	F	M	A	V	Q	W	A	G	G	P	I	I	L	T	D	OPX94088.1	3E-10	pfkB family carbohydrate kinase [Syntrophorhabdus sp. PtaB.Bin006]	Proteobacteria
97	K	R	R	F	M	D	V	Q	R	C	I	G	S	M	M	V	T	D	OPZ25350.1	3E-10	pfkB family carbohydrate kinase [Lentisphaerae bacterium ADurb.BinA184]	Lentisphaerae
111	K	R	R	F	M	G	V	Q	L	G	G	S	P	Y	A	M	M	D	OQB77460.1	3E-08	pfkB family carbohydrate kinase [Deltaproteobacteria bacterium ADurb.Bin135]	Proteobacteria

The ID numbers correspond to the order of the E-values in 143 homologs found by the BLAST search, which include the 52 proteins possessing the five key residues (No. 0 is the submitted sequence, the sequence of No. 1 displayed the smallest E-value, and the sequence of No. 143 displayed the largest E-value). Conserved residues in the donor-binding site are highlighted in blue or gray. Residues that are significantly different from TM0415 in the acceptor-binding site (**Supplementary Figure 4**) are highlighted in green. Domains of life are written in parentheses if the bacterial phylum is not defined.

Supplementary Table 3. Base sequence of the synthesized TM0415 gene.

Name	Sequence
TM0415	5'-CCATGGGATCTGATAAAATTCATCATCATCATCATCACATGATCACCTTCATTG<br>GGCATGTATCGAAAGACGTCAACGTGGTAGATGGAAAGAGGGAGATCGCGTAC<br>GGTGGGGGAGTGGTGATGGGGGCCATCACCTCCTCGCTGCTCGGTGTGAAAAC<br>AAAAGTGATTACAAAATGCACGAGAGAGGACGTTTCAAAGTTTTCTTTTCTCCG<br>AGACAATGGTGTGGAAGTGGTGTTTCTGAAAAGTCCTAGAACAACCAGCATTG<br>AGAACAGGTACGGATCAGATCCTGACACGAGGGAGAGTTTTTTGATATCGGCGG<br>CGGATCCGTTCACTGAAAGTGACCTGGCATTCATCGAAGGAGAGGCGGTTCATA<br>TCAACCCGCTCTGGTATGGAGAGTTTCCGGAGGATCTCATACCTGTTTTGCGAA<br>GGAAAGTGATGTTTCTCTCTGCCGACGCGCAGGGGTTTGTAAGAGTGCCAGAA<br>AATGAAAAACTCGTTTACAGAGACTGGGAGATGAAAGAGAAATATTTGAAGTA<br>CCTCGATCTCTTCAAGGTGGACAGCAGGGAAGCAGAAACACTCACGGGAACGA<br>ACGACTTGAGAGAGTCGTGCAGGATCATCCGTTCTTTTGGTGCGAAGATCATTC<br>TGGCAACACATGCGAGCGGTGTGATAGTCTTCGATGGAAACTTCTACGAAGCTT<br>CATTCAGAAGTTGGTCACTGGAAGGTAGAACGGGAAGAGGGGACACCTGTACT<br>GCGGCGTTTCTCGTTGGATTCGTCTTCAAAAAGATGAGCATCGAGAAGGCAACA<br>AAATTCGCGGCTGCTGTAACTTCTGTAAAGATGAGACATCCTGGACCACTGAGG<br>AGGGAGGATCTTGAAGCTATCTCTGGTGATCAGTACTTCTAACCATGG-3'

Restriction sites used for subcloning are underlined.

Supplementary Table 4. Oligonucleotides used for preparation of the TM0415 plasmids.

**Oligonucleotide**	**Sequence**
K171A-F4	5'-GATCTCTTCGCGGTGGACAGCAGGGAA-3'
K171A-R4	5'-CCACCGCGAAGAGATCGAGGTACTTCAA-3'
R229A-F	5'-GAAGGTGCAACGGGAAGAGGGGACAC-3'
R229A-R	5'-TCCCGTTGCACCTTCCAGTGACCAACT-3'
R232A-F	5'-ACGGGAGCAGGGGACACCTGTACTGC-3'
R232A-R	5'-GTCCCCTGCTCCCGTTCTACCTTCCAG-3'
tag-rm-F	5'-ATATACCATGATCACCTTCATTGGG-3'
tag-rm-R	5'-GTGATCATGGTATATCTCCTTCTTAA-3'

Supplementary Table 5. Base sequences of the synthesized genes of the TM0415 homologs.

Name	Sequence
Homolog No. 7	5’-CATATGGAAGAGAATCAGCAGCAATGCCCCGTACGGCACATTTGGGAGCCTCAACAAGGGCAGGTTCAAC<br>AACAGGCGAGTACTGGCAGCGAGTGCCCAGTCCGTAACACTTGGGACACCTCAAGCCTGTACCACACGAAC<br>GGTCAGTTCCAGCACCCGCCCATCTCTCAAACGAATGGCCAGGCGAGATGCCCCATGGGCTACTTCTCCATC<br>TCTGCTTCTTCGGGCGCTCCCGCCTCTTCTTCGGACGGTGCCCCCAAGTGCCCATTCGCGTCCCTCTCCCTGG<br>GcTCCAGCACAGACAGGAGGGCGCCCCCTGAGCAGCTGCGTCTGACCATTCTGGGCCACGTCACCAACGAC<br>ATCAACATCTTCGTCGGCAAGGAGACGCGCGCGCAGGGTGGTGGAGTGCTGTTCAGCGGTGTGGCTGCCTC<br>CAACTTGGGCATGAACGTTGAGGTTGTTACCAAGTGCTCGGCCGAGGACAAGCCCGTGTTCCAGAAGATCT<br>TTGCCCCCAGTGGAACCAAGGTACAGTTCCTGCCGTCGCAAGAGACGACTTGCTGTGAAAACAACTACCCC<br>AAGCCCAACTCGGATGAAAGAGTCCAACGCTTCCACGCTGTTGGCGCTCCCTTCACGGTGGACGATCTGAA<br>GCACATTGAATCCACAATCGTCCACATCAACCCTCTTTCATAcGGCGAGTTCCCTGACGAGCTGATCCCCAAG<br>ATCAAGGAGCTCAACCCCTCTGTTCAGTACCTGGTCGCCGACGCGCAGGGATTCATCAGGCACATCGACATG<br>CAGAACGGCGGCAAGATCTCGCACAAGGACTGGGCTGCCAAGGAGCAGTACCTCAAATACTTTGATTTGTTC<br>AAGGTGGACGACAAGGAGGCCACTGTCTTGACCGGAGAGAAGGACATGAAGCGCGCCATGCAGATTCTGC<br>ACGAGAAGGGCGCCAAGATGGTGCTCGGGACGTACAACGCGGGCGTGCTGTTGTTTGATGGCAACATCTTCT<br>ACCAGGCAAGCTTCGGTCCTTGGAAGGTAGAGGGAAGAACTGGCAGGGGAGATACTGTCACCGCGAGCTTC<br>CTTGCCGCCGCCGGGTTGAGCGGAGGACCCTGGAAGCGCAACGTCGCGCTTGAGTTTGCCGCCCGCGTGAC<br>CACCACCAAGATGCAGTACCCGGGTCCGTACCGTCGGCCAACGGCCTCGCTTTGAGGATCC-3’
Homolog No. 48	5’-CATATGAATAGATATGATCTTCTGTTTGTGGGCCATGTAACTATTGACGAAATCGAGGCAAAGGAAGGATCT<br>GCCCGTAGTGTGCCCGGTGGCGCACCGTTTTTCGGGGCTTTAGCCGCCTCTCGTAGCGGCAAGAGGATCGCC<br>GTGGTTACGAGAATGGCCAAAGAAGATGAAGGTTACCTTGCACTGTTGAAAGATGCCGGTATAGACATCTAC<br>CTGCAACCGGTCGCCCAGACTACCCACATGCGGGTTGTCCATCCCACAGAGAATGTGGACGAAAGGCTGAT<br>GTATCAGACACAGAACGCCGGGTTCTTTTCCCTGGAAGATCTATCCCCCGTACAGCCGTGCCCGGCTCACCTT<br>GGTGCCCTGACCGACCGGGAATTTACCCTGGGATTCATGCGAAGATTGAAGGAACGGGGTTTTCGGCTGTCA<br>ATAGATATGCAGAATTTTGTGCGCCAGGTTGACATGGAGACCGGTGTGATTCATTTCAAGGATGTACCGGAGA<br>AAAGAGAGATTGTGAGTCTCGCCGACATGGTAAAACTCGATGTGGTGGAAGCAGAAATACTCACCGGTACT<br>GACGATCTGGAGCAAGCCGCCGTGATTGTAGAAAAATGGGGATGTCCGGAGATCATCATAACCCGTTCAGAC<br>GGAGTTCTCGCCCGTTATAAGGGCAAAATGTACTTTGAAAAATTCTCTAACAGGAATTCTCAGGGCAGAACC<br>GGCCGTGGTGACACAACGACAGGGTCATATCTGGTCCGCAGGCTGGATCATGAGGTAGAAGACTCGTTAAAA<br>TTCGCAGCAGCCCTGGCTTCGATCAAGATGGAGACCCCCGGTCCCTTCAACGGAACCCTCGAAGATGTTCTT<br>AGAAGAATGGGCTCCTGAGGATCC-3’
Homolog No. 49	5’-CATATGGCGAATCAGCAGTCGGCTTACGACGTGGTTTTTATTGGTAATTACACCAAAGATACGATCATCACC<br>CCCGCGGGCACGCGCTACGTGGACGGCGGTGGGATGAACTATGCGGCCAACGCCGCGGCGCGCCTGGGCCT<br>GAAAACGGCGGTGGTCACGCGCCTATCGCGCGAAGATGTGCATGTGGTGGAGGGACTGCAGGCCAACGGG<br>GTGGATTGCTTTGCCACCTACAGCCCTTCTTCGACCCTGATGAAGCTGGAATACCCCACCACCGACCCCGAC<br>ATCCGCACCCTGACGGTGGCCGGGATCGCGGGCTCTATCACTGCCGCCGATGTGGACGGCTTTACCACCCGC<br>GCCGCGGCCATCAATTCCAGCCTGCGCGGGGAGGTGGGTCTGGATGTGATCCGCGCGCTGCGCGAGCGCGG<br>CACGCTGGTGGCGGCGGATATGCAGGGCTTTGTGCGCGTGCTGCGCGGCCAGAGCCTGATCTATGAACCCTG<br>GCCGGAGATGGAAGCCACCCTGGCCCAGGTGGACGTGGTGAAGAGCGACGCCGTGGAAGCGGAGTTCCTG<br>ACCGGCACCAAGGATATTTATCAGGCGGCCAAGATCTACGCGCAGATGGGCCCGGCTGAGATCGTTCTGACG<br>CATAAAGACGGCGTGCTGATCTACGACCGCGGTGCGACCTATGAATACGGCTTTTACCCGGCGCAATTGGTG<br>GGGCGCAGCGGGCGCGGCGATACCTGCGTGGGCACGTACCTTTCCAAGCGCCTGTCCATGAACCCGCAGGA<br>AGCTGGTTTGTGGGCGGCAGCCGTGACCAGCCTGAAGATGGAAGCCCTGGGCCCCTTCAACCGCTCGGTGC<br>CTGAGGTGGAGGCCTTTATCGCCAGTAAATACCGCCAGACGGTGGGCTAAGGATCC-3’
Homolog No. 92	5’-CATATGAATCGGTATGATCTTGTATTTATGGGTCATTTGGCGACAGCCATCATTGTTCCTTTCGAGGGCCCCC<br>CTTTCATCGAACGCGGTGGCCCGGCTTTCTTCGGTCCCATAGCAGCCTCCTGTTTGACAAGAAGGATTGCTG<br>CAGTGACGAGCATTGCGGAGAATGAAGCACAGCTTTTGGAACCGCTGCAAGCTGCCGGCATCGATCTCTTCA<br>TGCAACCTCGAGAAACTGCCCAAATGCGGGTCGTCCAGCCTAGCCGGAATGTCGATGAGAGGCAGATTTTTC<br>TCACAAAGCGCGGAGGATGCTTTTGCGCAGGCGACATTCCCCCTATCGATCCGTGTCTGATCCACCTTGGCG<br>GCCTGAGCGATCACGAGTTTACCCTGGAATTCATGCGGGCGCTCAAAGCGCGCGGATTCCGTTTGTCGGTGG<br>ATATACAGAGTTTTGTCTGGCACGTAGACGATCGGACGCAGCTTATTCACTGGGAGGATATACCGGAAAAAC<br>ACGACATCCTGACCATGGTCGATTTCATAAAGCTTGATGTAAAAGAAGCAGCCACGCTGACCGGTACCAGCG<br>TTCTCCATGAACAGGCGGAAATACTGGAAGGATGGGGAAGCTCCGAGACTGTAATAACGTGTTCAAAGGGG<br>GCACTGGCACGCAACAAGGGGAAAACCACTTTCACACGGTTCACCAACAGGAGCACCCAGGGGAGAACCG<br>GCCGCGGCGATACCTTTTCCGGGGCATACCTTGCCCGGAGGCTGGATCACTCCGTCGAAGAGTCTCTGAAAT<br>TTGCCGCAGCACTGACCTCCATCAAGATGGAATCCGTCGGCCCCTTCAGGGGCTCTCTCGAGGATGTTATCA<br>AGAGAATGGGCAACTCCCTTTCTCCTTGAGGATCC-3’
Homolog No. 111	5’-CATATGAGCAAGAAGAACCAGTATGATATTGTGTTCGTGGGACAGATGGGTAGGGGCACTGTAGTTCCTTT<br>TGAAGGAGTTCCCTTCGTTATATTGGGCAGCCCGGTTCTGTTTGCCTCAATAGCAGCATCTTGTGTTGGAAAG<br>AGGATTGCTGTGGTTACGACAATCTCCAAAAAGGAAGAATACCTTTTGGAGCCTATGAAAAAGGCTGGTATA<br>GATCTCTACATTCAGCCTGGAGAGACTGCTCAATACCGTGTTGTCTTCCCAAATGCAAATGTTGATGAGAGGC<br>AGGCTTTTCATGTTAAAGGGGGAAGTAATATTGAAAAAGTACCCCCTTTTGAGCCATGCCTGTTGCACTGTTG<br>CTGTATGGGCCCTCGCGAGGTCCAGATGGATTTGATGAGGTCACTAAAGGCAAGAGGATTCCGTTTATCAGT<br>AGACATGCAGGGTCTTATGCTGCAGGCAGACCGTGAGACCGGCATTGTCCGCCTTGAAGATTTTCCAGAGAA<br>AAAAGAAATCCTGAGAATGGCAGATTTCGTAAAGCTTGACAGTAAGGAGGCGCAGGCTTTGACAGGCACCG<br>ACGTCTTACAAGACCAGGCTGCCATACTGGAAGGCTGGGGAAGCCCTGAGACTATTATTACGTCTTCAGGTG<br>GTGTACTGGCGCGAAGCCACGGAACAACAAAATATGCAAAATTTTCAAACAGGAGCACTAAAGGCAGGATG<br>GGGCGAGGCGACACGGTCATCGGATCTTATATAGCGCGCAGGTTTGATTATCCTGTTGAAGACTCTCTTCAGT<br>TCGCTGGAGCCCTTGCATCGATCAAGATGGAGTCTGTTGGGCCATTTGCGGGCTCCCTGGAAGATGTGATTG<br>AGAGAATGGGTGATTCAGTGCCATGTCAATCAATCTAAGGATCC-3’

Restriction sites used for subcloning are underlined.

Supplementary Table 6. Crystallographic data and refinement statistics.

	PCP complex*	SO_4^{2-} complex*
**Data collection**		
Space group	$P2_1$	$P2_1$
Cell dimensions		
a, *b*, *c* (Å)	46.7, 63.0, 89.9	46.8, 62.4, 88.0
β (°)	105.1	104.0
Resolution (Å)	50–1.70 (1.73–1.70) **	50–1.47 (1.50–1.47)
R_{sym}	0.045 (0.505)	0.039 (0.394)
$I / \sigma I$	24.8 (2.1)	27.8 (3.1)
Completeness (%)	98.1 (96.7)	95.7 (94.3)
Redundancy	3.3 (3.2)	3.4 (3.3)
**Refinement**		
Resolution (Å)	50–1.70 (1.73–1.70)	50–1.47 (1.50–1.47)
No. reflections	51566	75813
R_{work} / R_{free}	0.1964/0.2104	0.1931/0.2045
No. atoms		
Protein	4168	4504
Ligand	34	24
Solvent	100	278
B-factors (Å^2)		
Protein	35.1	18.2
Ligand	32.2	15.8
Solvent	31.5	19.7
R.m.s. deviations		
Bond lengths (Å)	0.0099	0.0145
Bond angles (°)	1.3823	1.6952
Ramachandran plot		
Favored (%)	99.3	99.1
Allowed (%)	100	100

*One crystal was used for each structure.

**Values in parentheses are for highest-resolution shell.

Supplementary Table 7. Abbreviations of the ribokinase family enzymes.

Abbreviation	Description	PDB ID	Reference
MI3K_TK	*myo*-inositol 3-kinase from *Thermococcus kodakarensis*	4XF7	6
NK_MJ	nucleoside kinase (NK) from *Methanocaldococcus jannaschii*	2C49	7
NK_BT	NK from *Burkholderia thailandensis*	3B1Q	8
AK_MT	adenosine kinase (AK) from *Mycobacterium tuberculosis*	2PKK	9
AK_TG	AK from *Toxoplasma gondii*	1LII	10
AK_AG	AK from *Anopheles gambiae*	3LOO	11
AK_TB	AK from *Trypanosoma brucei rhodesience*	3OTX	12
AK_HS	AK from *Homo sapiens*	1BX4	13
RK_EC	ribokinase (RK) from *E. coli*	1RK2	14
RK_HS	RK from *H. sapiens*	2FV7	15
RK_SA	RK from *Staphylococcus aureus*	3RY7	16
KDGK_SS	2-keto-3-deoxygluconate kinase (KDGK) from *Sulfolobus solfataricus*	2VAR	17
KDGK_TT	KDGK from *Thermus thermophilus*	1V1A	18
KDGK_TM	KDGK from *T. maritima*	2AFB	19
T6PK_SA	D-tagatose-6-phosphate kinase from *S. aureus*	2JG1	20
FK_HS	fructokinase from *H. sapiens*	2HW1	21
AIRK_SE	aminoimidazole riboside kinase from *Salmonella enterica*	1TZ6	22
HldA_BC	D-glycero-β-D-manno-heptose 7-phosphate kinase from *Burkholderia cenocepacia*	4E8Y	23
PFK2_EC	phosphofructokinase-2 from *E. coli*	3UQD	24
PK_TB	pyridoxal kinase (PK) from *Trypanosoma brucei*	3ZS7	25
PK_OA	PK from *Ovis aries*	1RFU	26
PK_EC1	PK from *E. coli*	1VI9	27
PK_HS	PK from *H. sapiens*	3FHY	28
PK_BS	PK from *Bacillus subtilis*	2I5B	29
PK_EC2	PK from *E. coli*	2DDO	30
HMPPK_ST	4-amino-5-hydroxymethyl-2-methylpyrimidine phosphate kinase from *Salmonella typhimurium*	1JXI	3
THZK_BS	4-methyl-5-β-hydroxyethylthiazole kinase from *B. subtilis*	1EKQ	31
AGK_PF	ADP-dependent glucokinase (AGK) from *P. furiosus*	1UA4	32
AGK_TL	AGK from *T. litoralis*	4B8S	33
APFK_PH	ADP-dependent phosphofructokinase from *Pyrococcus horikoshii*	1U2X	34

Supplementary References

1. Rodionova, I.A. *et al.* Novel inositol catabolic pathway in *Thermotoga maritima*. *Environ. Microbiol.* **15**, 2254-2266 (2013).
2. Andersson, C.E. & Mowbray, S.L. Activation of ribokinase by monovalent cations. *J. Mol. Biol.* **315**, 409-419 (2002).
3. Cheng, G., Bennett, E.M., Begley, T.P. & Ealick, S.E. Crystal structure of 4-amino-5-hydroxymethyl-2-methylpyrimidine phosphate kinase from *Salmonella typhimurium* at 2.3 Å resolution. *Structure* **10**, 225-235 (2002).
4. Davis, I.W. *et al.* MolProbity: all-atom contacts and structure validation for proteins and nucleic acids. *Nucleic Acids Res.* **35**, W375-W383 (2007).
5. Chen, V.B. *et al.* MolProbity: all-atom structure validation for macromolecular crystallography. *Acta Crystallogr. D Biol. Crystallogr.* **66**, 12-21 (2010).
6. Nagata, R., Fujihashi, M., Sato, T., Atomi, H. & Miki, K. Crystal structure and product analysis of an archaeal *myo*-inositol kinase reveal substrate recognition mode and 3-OH phosphorylation. *Biochemistry* **54**, 3494-3503 (2015).
7. Arnfors, L., Hansen, T., Schönheit, P., Ladenstein, R. & Meining, W. Structure of *Methanocaldococcus jannaschii* nucleoside kinase: an archaeal member of the ribokinase family. *Acta Crystallogr. D Biol. Crystallogr.* **62**, 1085-1097 (2006).
8. Yasutake, Y., Ota, H., Hino, E., Sakasegawa, S. & Tamura, T. Structures of *Burkholderia thailandensis* nucleoside kinase: implications for the catalytic mechanism and nucleoside selectivity. *Acta Crystallogr. D Biol. Crystallogr.* **67**, 945-956 (2011).
9. Reddy, M.C. *et al.* High resolution crystal structures of *Mycobacterium tuberculosis* adenosine kinase: insights into the mechanism and specificity of this novel prokaryotic enzyme. *J. Biol. Chem.* **282**, 27334-27342 (2007).
10. Schumacher, M.A. *et al.* Crystal structures of *Toxoplasma gondii* adenosine kinase reveal a novel catalytic mechanism and prodrug binding. *J. Mol. Biol.* **298**, 875-893 (2000).
11. Cassera, M.B. *et al.* A high-affinity adenosine kinase from *Anopheles gambiae*. *Biochemistry* **50**, 1885-1893 (2011).
12. Kuettel, S. *et al.* Crystal structures of *T. b. rhodesiense* adenosine kinase complexed with inhibitor and activator: implications for catalysis and hyperactivation. *PLoS Negl. Trop. Dis.* doi: 10.1371/journal.pntd.0001164 (2011).
13. Mathews, I.I., Erion, M.D. & Ealick, S.E. Structure of human adenosine kinase at 1.5 Å resolution. *Biochemistry* **37**, 15607-15620 (1998).
14. Sigrell, J.A., Cameron, A.D. & Mowbray, S.L. Induced fit on sugar binding activates ribokinase. *J. Mol. Biol.* **290**, 1009-1018 (1999).
15. Park, J., van Koeverden, P., Singh, B. & Gupta, R.S. Identification and characterization of human ribokinase and comparison of its properties with *E. coli* ribokinase and human adenosine kinase. *FEBS Lett.* **581**, 3211-3216 (2007).

16. Li, J. *et al.* Crystal structure of Sa239 reveals the structural basis for the activation of ribokinase by monovalent cations. *J. Struct. Biol.* **177**, 578-582 (2012).
17. Potter, J.A. *et al.* The structure of *Sulfolobus solfataricus* 2-keto-3-deoxygluconate kinase. *Acta Crystallogr. D Biol. Crystallogr.* **64**, 1283-1287 (2008).
18. Ohshima, N., Inagaki, E., Yasuike, K., Takio, K. & Tahirov, T.H. Structure of *Thermus thermophilus* 2-keto-3-deoxygluconate kinase: evidence for recognition of an open chain substrate. *J. Mol. Biol.* **340**, 477-489 (2004).
19. Mathews, I.I. *et al.* Crystal structure of 2-keto-3-deoxygluconate kinase (TM0067) from *Thermotoga maritima* at 2.05 Å resolution. *Proteins* **70**, 603-608 (2008).
20. Miallau, L., Hunter, W.N., McSweeney, S.M. & Leonard, G.A. Structures of *Staphylococcus aureus* d-tagatose-6-phosphate kinase implicate domain motions in specificity and mechanism. *J. Biol. Chem.* **282**, 19948-19957 (2007).
21. Trinh, C.H., Asipu, A., Bonthron, D.T. & Phillips, S.E. Structures of alternatively spliced isoforms of human ketohexokinase. *Acta Crystallogr. D Biol. Crystallogr.* **65**, 201-211 (2009).
22. Zhang, Y., Dougherty, M., Downs, D.M. & Ealick, S.E. Crystal structure of an aminoimidazole riboside kinase from *Salmonella enterica*: implications for the evolution of the ribokinase superfamily. *Structure* **12**, 1809-1821 (2004).
23. Lee, T.W. *et al.* Structural-functional studies of *Burkholderia cenocepacia* D-glycero-β-D-manno-heptose 7-phosphate kinase (HldA) and characterization of inhibitors with antibiotic adjuvant and antivirulence properties. *J. Med. Chem.* **56**, 1405-1417 (2013).
24. Cabrera, R., Ambrosio, A.L., Garratt, R.C., Guixé, V. & Babul, J. Crystallographic structure of phosphofructokinase-2 from *Escherichia coli* in complex with two ATP molecules. Implications for substrate inhibition. *J. Mol. Biol.* **383**, 588-602 (2008).
25. Jones, D.C., Alphey, M.S., Wyllie, S. & Fairlamb, A.H. Chemical, genetic and structural assessment of pyridoxal kinase as a drug target in the African trypanosome. *Mol. Microbiol.* **86**, 51-64 (2012).
26. Li, M.H. *et al.* Conformational changes in the reaction of pyridoxal kinase. *J. Biol. Chem.* **279**, 17459-17465 (2004).
27. Badger, J. *et al.* Structural analysis of a set of proteins resulting from a bacterial genomics project. *Proteins* **60**, 787-796 (2005).
28. Gandhi, A.K. *et al.* Kinetic and structural studies of the role of the active site residue Asp235 of human pyridoxal kinase. *Biochem. Biophys. Res. Commun.* **381**, 12-15 (2009).
29. Newman, J.A., Das, S.K., Sedelnikova, S.E. & Rice, D.W. The crystal structure of an ADP complex of *Bacillus subtilis* pyridoxal kinase provides evidence for the parallel emergence of enzyme activity during evolution. *J. Mol. Biol.* **363**, 520-530 (2006).
30. Safo, M.K. *et al.* Crystal structure of pyridoxal kinase from the *Escherichia coli pdxK* gene: implications for the classification of pyridoxal kinases. *J. Bacteriol.* **188**, 4542-4552 (2006).
31. Campobasso, N., Mathews, I.I., Begley, T.P. & Ealick, S.E. Crystal structure of 4-methyl-5-β-hydroxyethylthiazole kinase from *Bacillus subtilis* at 1.5 Å resolution. *Biochemistry* **39**,

7868-7877 (2000).

32. Ito, S. *et al.* Crystal structure of an ADP-dependent glucokinase from *Pyrococcus furiosus*: implications for a sugar-induced conformational change in ADP-dependent kinase. *J. Mol. Biol.* **331**, 871-883 (2003).
33. Rivas-Pardo, J.A. *et al.* Crystal structure, SAXS and kinetic mechanism of hyperthermophilic ADP-dependent glucokinase from *Thermococcus litoralis* reveal a conserved mechanism for catalysis. *PLoS One* doi: 10.1371/journal.pone.0066687 (2013).
34. Currie, M.A. *et al.* ADP-dependent 6-phosphofructokinase from *Pyrococcus horikoshii* OT3: structure determination and biochemical characterization of PH1645. *J. Biol. Chem.* **284**, 22664-22671 (2009).
